# Tick-Borne Relapsing Fever in the White Mountains, Arizona, USA, 2013–2018

**DOI:** 10.3201/eid2504.181369

**Published:** 2019-04

**Authors:** Neema Mafi, Hayley D. Yaglom, Craig Levy, Anissa Taylor, Catherine O’Grady, Heather Venkat, Kenneth K. Komatsu, Brentin Roller, Maria T. Seville, Shimon Kusne, John Leander Po, Shannon Thorn, Neil M. Ampel

**Affiliations:** Mayo Clinic, Phoenix, Arizona, USA (N. Mafi, M.T. Seville, S. Kusne, N.M. Ampel);; Arizona Department of Health Services, Phoenix (H.D. Yaglom, H. Venkat, K.K. Komatsu);; Maricopa County Department of Public Health, Phoenix (C. Levy);; Pima County Health Department, Tucson, Arizona, USA (A. Taylor, C. O’Grady);; Centers for Disease Control and Prevention, Atlanta, Georgia, USA (H. Venkat);; University of Arizona College of Medicine, Tucson (B. Roller, J.L. Po, N.M. Ampel); Northwest Hospital, Tucson (S. Thorn)

**Keywords:** ticks, relapsing fever, zoonoses, public health, Arizona, United States, TBRF, tickborne diseases, Borrelia hermsii, bacteria, bacterial infections, White Mountains, vector-borne infections, tick-borne relapsing fever

## Abstract

Tick-borne relapsing fever (TBRF) is a bacterial infection transmitted by tick bites that occurs in several different parts of the world, including the western United States. We describe 6 cases of TBRF acquired in the White Mountains of Arizona, USA, and diagnosed during 2013–2018. All but 1 case-patient had recurrent fever, and some had marked laboratory abnormalities, including leukopenia, thrombocytopenia, hyperbilirubinemia, and elevated aminotransaminases. One patient had uveitis. Diagnosis was delayed in 5 of the cases; all case-patients responded to therapy with doxycycline. Two patients had Jarisch-Herxheimer reactions. The White Mountains of Arizona have not been previously considered a region of high incidence for TBRF. These 6 cases likely represent a larger number of cases that might have been undiagnosed. Clinicians should be aware of TBRF in patients who reside, recreate, or travel to this area and especially for those who sleep overnight in cabins there.

The White Mountains comprise a relatively dry, high-elevation region in the eastern part of Arizona that is in close proximity to Phoenix and Tucson, the 2 major metropolitan areas of Arizona, both of which are in lower-elevation deserts. Ranging from 6,000 feet (1,829 m) to >11,000 feet (>3,353 m) in elevation, the White Mountains are a place for recreation and respite for urban dwellers, particularly during the hot summer months, and many cabins and summer homes are located throughout the region. Persons who acquire illnesses in the White Mountains often seek medical care in the desert metropolitan communities, and physicians there might not be aware of the differences in the diseases or their host habitats in these mountains compared with the lower-elevation deserts.

Tick-borne relapsing fever (TBRF) is a bacterial infection transmitted by tick bites. In the United States, TBRF most often occurs in western states and is usually transmitted by bites of *Ornithodoros* spp. ticks; *Borrelia hermsii* is thought to be the most common cause. We describe 6 cases of TBRF occurring over a 6-year period in persons who visited the White Mountains. Although infection probably was acquired in these mountains, given the timeframe of exposure to illness onset (all had plausible exposure within the ≈7-day incubation period), all cases were diagnosed in either Phoenix or Tucson. We suspect that these cases represent a small proportion of the total number of cases and would alert both clinicians and public health officials to the presence of TBRF in this area.

## The Case-Patients

### Case-Patient 1

A 34-year-old man with no prior medical history had fevers in early July 2013. One week before fever onset, he had spent time with his family in a privately owned cabin in Alpine, Arizona, in the White Mountains. Mice and ticks had been seen in the cabin, but the patient was not aware of any bites. Fevers reaching 39.9°C with chills, headache, arthralgias, and myalgias were reported. These symptoms persisted for ≈3 days, remitted for 3 days, and then recurred for the next 3.5 weeks. Laboratory evaluation of the patient 2 weeks after symptom onset was notable for mild anemia and markedly elevated C-reactive protein but normal leukocyte and platelet counts and aminotransaminases. After ≈25 days, the fevers stopped, but the patient noticed floaters and blurry vision in his right eye. In August, he was seen by a retinal expert in Phoenix, and anterior and posterior uveitis with optic nerve inflammation were noted. A Lyme ELISA serologic screening test result was positive, and Western blot revealed IgM bands of 23 and 41 kDa and negative IgG. A Lyme-specific rapid plasma reagin test was negative. An infectious diseases consultant recommended treatment for possible TBRF, and oral doxycycline (100 mg 2×/d) for 14 days was prescribed. The patient had complete resolution of his visual symptoms 2 months after completing antimicrobial therapy. Immunofluorescent serologic tests for *B. hermsii* conducted 9 weeks after symptom onset demonstrated an IgM titer of 1:64 and an IgG titer of 1:512.

### Case-Patient 2

**Figure 1:**
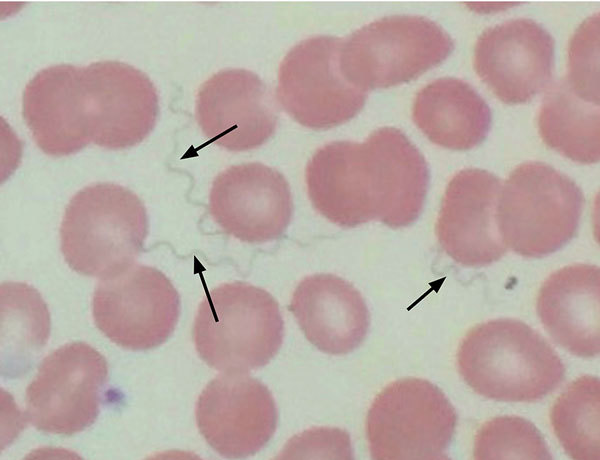
Wright stain of peripheral blood demonstrating extracellular spirochetes (arrows) confirming tick-borne relapsing fever in a 64-year-old woman, Tucson, Arizona, USA, October 2016.

A 64-year-old woman with a medical history of hypothyroidism and hypertension sought care at an emergency department in Tucson in late October 2016 because of a 10-day history of fevers with rigors, fatigue, body aches, and generalized malaise. The symptoms began on October 24, one day after she returned from her cabin in the White Mountains, where she had spent 10 days with her husband doing various recreational activities. The patient denied insect bites or stings. Physical examination revealed a well-appearing female in no acute distress with mild resting tachycardia, normal temperature, and right upper quadrant tenderness to palpation. Laboratory values were remarkable for a total leukocyte count of 2,900 cells/µL with normal differential, platelet count of 32,000/µL, total bilirubin of 2.3 mg/dL, alkaline phosphatase of 483 IU/L, alanine aminotransferase of 78 IU/L, and aspartate aminotransferase of 137 IU/L. The patient was admitted to the hospital, and a subsequent Wright-stain peripheral blood smear demonstrated numerous spirochete-like organisms on light microscopic examination (Figure 1). A Lyme disease screening test was positive. A diagnosis of TBRF was made based on the positive blood smear result with matching clinical picture, and the patient was prescribed oral doxycycline (100 mg 2×/d) for 10 days. Within a few hours of receiving the first dose of doxycycline, she became hypotensive and hypoxic and required a 24-hour stay in the medical intensive care unit for supportive care. After her intensive care unit stay, she had a rapid and complete resolution of her symptoms and normalization of her laboratory values. Serum sent to the Centers for Disease Control and Prevention (CDC; Fort Collins, CO, USA) for Western blot assay against *B. hermsii* showed strongly positive results for both IgM and IgG.

### Case-Patient 3

A 72-year-old man with a medical history of hyperlipidemia and complete heart block requiring a permanent pacemaker spent ≈3 weeks at his cabin in the White Mountains near Greer, Arizona, doing outdoor cleaning in early June 2017. On June 22, he had onset of shaking chills and sought evaluation at a local emergency department, where sinusitis was diagnosed and amoxicillin prescribed. A temperature of 38.1°C was recorded. Several hours after taking a single dose of amoxicillin, he experienced severe rigors, prompting him to return to the emergency department. He was admitted to the hospital, where peripheral leukocyte count was 1,800 cells/µL, platelet count was 44,000/µL, total bilirubin was 2.3 mg/dL, and alanine aminotransferase was 78 IU/L. A peripheral blood smear demonstrated toxic granules in the neutrophils but no organisms. The patient improved without further antimicrobial therapy and was discharged home. One week later, he presented to an emergency department in Phoenix with recurrent fevers, fatigue, and rigors. A peripheral blood leukocytosis with lymphopenia was observed over several days. Because of positive coccidioidal serologic results, fluconazole was started; the patient was discharged home after his fevers resolved during hospital observation. He was seen 2 weeks later after a third episode of fever with rigors and was prescribed oral doxycycline (100 mg 2×/d). Within hours of the first doxycycline dose, he developed rigors, dizziness, and tachycardia and discontinued treatment. Subsequently, a PCR test for *B. miyamotoi* on whole blood yielded an indeterminate result. Doxycycline was restarted without further adverse effects, and the patient completed a 2-week course. Immunofluorescent serologic testing for *B. hermsii* demonstrated an IgG titer of 1:128. Serum sent to CDC for tickborne diseases was found to be positive by Western blot assay for *B. hermsii* IgM and IgG. The patient later recalled a possible insect or tick bite on his forearm during his stay at his cabin.

### Case-Patient 4

A previously healthy 4-year-old boy stayed in a cabin near Greer during the last week of May and the first week of June 2017 and experienced 1 month of recurring fever, chills, sweats, body aches, and headaches beginning on June 5. Blood samples sent to CDC at 28 and 43 days after symptoms started demonstrated *B. hermsii* IgM and IgG by Western blot assay. The patient completed a course of doxycycline and completely recovered. During the investigation, local and state public health authorities learned of >6 other family members who visited the same cabin over a 2-year period, and all reported febrile illnesses. Only 1, case-patient 5, was tested for and diagnosed with TBRF.

### Case-Patient 5

A 41-year-old man stayed in the same cabin as case-patient 4 from late May through early June 2017. On June 2, the man had onset of fever reaching 39.4°C with sweats, chills, headaches, fatigue, and dizziness. He visited healthcare providers multiple times during the month of June for evaluation of these symptoms. ELISA and Western blot tests for Lyme disease and coccidioidomycosis were positive. Finally, on August 30, ≈12 weeks after symptoms began, a serum sample sent to CDC was positive by Western blot assay for *B. hermsii* IgM and IgG. The patient was treated with doxycycline (100 mg 2×/d), and his symptoms resolved.

### Case-Patient 6

A 71-year-old woman with a medical history of hypertension, hypothyroidism, and past splenectomy for idiopathic thrombocytopenic purpura had sudden onset of fever, chills, generalized weakness, and myalgias in late July 2018, four days after visiting her family-owned cabin in Alpine. She subsequently sought care at an emergency department in Tucson. She had a temperature of 38.9°C and was found to be tachypneic, hypotensive, and hypoxemic. She was admitted to the intensive care unit, where it was noted that her initial peripheral leukocyte count was 11,400 cells/µL, platelet count was 39,000/µL, and serum creatinine was 2.7 mg/dL. Spirochetes were identified on peripheral blood smear by using light microscopic examination. During her hospital stay, the patient experienced acute respiratory failure and septic shock and required mechanical ventilation. A diagnosis of TBRF was made, and doxycycline (100 mg 2×/d) was initiated for a total of 10 days. The patient improved and was discharged home. She recalled substantial rodent activity in the area of the cabin. No other persons staying at the cabin reported illness.

## Discussion

Vector-associated illnesses are increasing in frequency across the United States (*1*). Compared with more common vectorborne illnesses such as Lyme borreliosis, Rocky Mountain spotted fever, tularemia, babesiosis, anaplasmosis, and ehrlichiosis, far less is known about TBRF, so much so that it has been described as a neglected disease (*2*).

TBRF typically manifests after a 7-day incubation period with recurring episodes of fever in association with headache, myalgias, and other nonspecific symptoms lasting for ≈3 days and separated by afebrile periods of ≈7 days’ duration. In the United States, *B. hermsii* is thought to be the most common cause of TBRF, and transmission is associated with the bite of soft *O. hermsi* ticks. *Ornithodoros* spp. ticks usually feed at night on rodents, such as tree squirrels and chipmunks, but might choose to feed on human hosts, particularly when the rodent population has been cleared from the local habitat. Feeding is rapid, and most persons who are bitten are not aware of a tick bite. Cabins are a typical site to acquire infection. In addition, soft ticks can live up to 10 years; this long lifespan means that once a cabin or building is infested, it can remain a source of human infection for years unless steps are taken to find and remove rodent infestations and eradicate the ticks. The risk for reinfection for any 1 person has not been calculated; however, repeated exposures, especially at the same sites as initial infection, are possible (*3*).

The 6 cases we describe occurred among persons inhabiting cabins in the White Mountains of Arizona during the late spring to early fall months. All but 1 person sought care with recurrent fever as their primary complaint; some had profound laboratory abnormalities, including leukopenia, thrombocytopenia, hyperbilirubinemia, and elevated aminotransaminases. One patient had organ-specific disease (uveitis). All case-patients responded promptly to doxycycline therapy; 2 had Jarisch-Herxheimer reactions in association with antimicrobial therapy, a phenomenon previously described (*4*). Two case-patients had positive serologic results for coccidioidomycosis, which were likely coincidental, given the relatively high incidence of this fungal infection in the desert regions of Arizona (*5*). The positive tests for Lyme borreliosis were likely attributable to cross-reactivity, as has been previously noted (*6*). 

**Figure 2:**
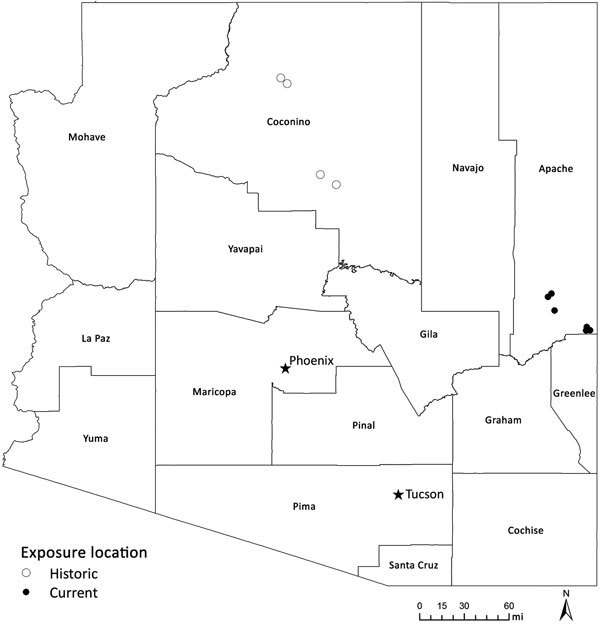
Exposure location for historic (1973–2014) tickborne relapsing fever outbreaks in Coconino County and 6 current (2013–2018) cases in the White Mountains region, Arizona, USA.

TBRF in Arizona has been described in the past around or near the Grand Canyon (*7*–*9*), several hundred miles from the site of these 6 cases (Figure 2). Apache County, the location of the 2013–2018 cases we have described, has been considered to be a comparatively low-incidence area for TBRF (*3*).

Unfortunately, no epidemiologic or environmental assessments were performed in these 6 cases. Although the benefits of conducting such studies are recognized, these studies were not performed here primarily because the cases were episodic. Limited resources of the local public health jurisdictions and lack of compliance from cabin owners also contributed. If future cases occur, such assessments would be useful to define whether *B. hermsii* is indeed the cause of the syndromes observed and to better define the ecologic, environmental, and epidemiologic parameters of the disease.

In Arizona, TBRF is a reportable disease by physicians to the local health department according to 2018 Arizona statutes (AR9-6-202). Although TBRF is not a nationally notifiable condition, prompt reporting of TBRF cases was required in >12 states in 2016 (Arizona, California, Colorado, Idaho, Montana, North Dakota, Nevada, New Mexico, Oregon, Texas, Utah, and Washington). Because of frequent travel by US residents, healthcare providers should report cases to the appropriate state or local health department; prompt reporting by clinicians has been critical to the identification and control of several outbreaks. For example, in 2014, a local Arizona hospital notified the Coconino County Public Health Services District (Flagstaff) of a cluster of 5 students with fever, headache, and myalgia who had attended the same camp; the ensuing investigation led to identification of an outbreak of 10 cases of TBRF among a high school football team who had attended an outdoor education camping trip in northern Arizona (*9*). 

The first case of TBRF in Arizona was reported in 1930, involving an exposure near Greer, similar to the 6 cases we have described. In that instance, after staying in the area for 18 days during the summer, a 37-year-old man had repeated bouts of fever with interval periods of well-being lasting several days. During 1 febrile attack, which occurred >1 month after symptoms began, a blood smear demonstrated spirochetes. The patient received an arsenial injection, and his fevers resolved (*10*).

Far more cases of TBRF probably have occurred recently in the White Mountains than the 6 reported here. Unlike previous recent reports from other parts of Arizona (*7*–*9*), the cases we report were not epidemic and therefore could have been easily missed. Therefore, physicians practicing in areas that serve this region should be aware of TBRF and consider the diagnosis when a patient has a nonspecific febrile illness, particularly when that illness is recurrent. In addition, all healthcare providers should assess travel history in patients who have nonspecific febrile illnesses and should consider diseases related to specific travel exposures. In addition to being able to recognize TBRF, healthcare providers in Arizona should be vigilant for other zoonotic diseases that could result from local exposures, such as hantavirus from deer mice, plague from fleas and prairie dogs, and Rocky Mountain spotted fever transmitted from brown dog ticks.
